# Minimal Circuit Model of Reward Prediction Error Computations and Effects of Nicotinic Modulations

**DOI:** 10.3389/fncir.2018.00116

**Published:** 2019-01-08

**Authors:** Nicolas Deperrois, Victoria Moiseeva, Boris Gutkin

**Affiliations:** ^1^Group for Neural Theory, LNC2 INSERM U960, DEC, École Normale Supérieure PSL* University, Paris, France; ^2^Center for Cognition and Decision Making, Institute for Cognitive Neuroscience, National Research University Higher School of Economics, Moscow, Russia

**Keywords:** dopamine, reward-prediction error, ventral tegmental area, acetylcholine, nicotine

## Abstract

Dopamine (DA) neurons in the ventral tegmental area (VTA) are thought to encode reward prediction errors (RPE) by comparing actual and expected rewards. In recent years, much work has been done to identify how the brain uses and computes this signal. While several lines of evidence suggest the interplay of the DA and the inhibitory interneurons in the VTA implements the RPE computation, it still remains unclear how the DA neurons learn key quantities, for example the amplitude and the timing of primary rewards during conditioning tasks. Furthermore, endogenous acetylcholine and exogenous nicotine, also likely affect these computations by acting on both VTA DA and GABA (γ -aminobutyric acid) neurons via nicotinic-acetylcholine receptors (nAChRs). To explore the potential circuit-level mechanisms for RPE computations during classical-conditioning tasks, we developed a minimal computational model of the VTA circuitry. The model was designed to account for several reward-related properties of VTA afferents and recent findings on VTA GABA neuron dynamics during conditioning. With our minimal model, we showed that the RPE can be learned by a two-speed process computing reward timing and magnitude. By including models of nAChR-mediated currents in the VTA DA-GABA circuit, we showed that nicotine should reduce the acetylcholine action on the VTA GABA neurons by receptor desensitization and potentially boost DA responses to reward-related signals in a non-trivial manner. Together, our results delineate the mechanisms by which RPE are computed in the brain, and suggest a hypothesis on nicotine-mediated effects on reward-related perception and decision-making.

## 1. Introduction

To adapt to their environment, animals constantly compare their predictions with new environmental outcomes (rewards, punishments, etc.). The difference between prediction and outcome is the prediction error, which in turn can serve as a teaching signal to allow the animal to update its predictions and render previously neutral stimuli predictive of rewards into reinforcers of behavior. Particularly, the dopamine (DA) neuron activity in the Ventral Tegmental Area (VTA) have been shown to encode the reward prediction error (RPE), or the difference between the actual reward the animal receives and the expected reward (Schultz et al., 1997; Schultz, 1998; Bayer and Glimcher, 2005; Day and Carelli, 2007; Matsumoto and Hikosaka, 2009; Enomoto et al., 2011; Eshel et al., 2015; Keiflin and Janak, 2015). During, for example, classical conditioning with appetitive rewards, unexpected rewards elicit strong transient increases in VTA DA neuron activity, but as a cue fully predicts the reward, the same reward produces little or no DA neurons response. Finally, after learning, if the reward is omitted, DA neurons pause their firing at the moment reward is expected (Schultz et al., 1997; Schultz, 1998; Keiflin and Janak, 2015; Watabe-Uchida et al., 2017). Thus DA neurons should either receive or compute the RPE. While several lines of evidence have pointed toward the RPE being computed by the VTA local circuitry, exactly how this is done vis-a-vis the inputs and how this computation is modulated by the endogenous acetylcholine and the endogenous substances that affect the VTA, e.g., nicotine, remains to be defined. Here we proceed to address these questions using a minimal computational modeling methodology.

In order to compute the RPE, the VTA should receive the relevant information from its inputs. Intuitively, distinct biological inputs to the VTA must differentially encode actual and expected rewards that are finally subtracted by a downstream target, the VTA DA neurons. For the last two decades, a great amount of experimental studies depicted which brain areas send this information to the VTA. Notably, a subpopulation of pedunculopontine tegmental nucleus (PPTg) has been found to send the actual reward signal to dopamine neurons (Kobayashi and Okada, 2007; Okada et al., 2009; Keiflin and Janak, 2015), while other studies showed that the prefrontal cortex (PFC) and the nucleus accumbens (NAc) respond to the predictive cue (Funahashi, 2006; Keiflin and Janak, 2015; Oyama et al., 2015; Connor and Gould, 2016; Le Merre et al., 2018), highly depending on VTA DA feedback projections in the PFC (Puig et al., 2014; Popescu et al., 2016) and the NAc (Yagishita et al., 2014; Keiflin and Janak, 2015; Fisher et al., 2017). However, how each of these signals are integrated by VTA DA neurons during classical-conditioning remains elusive.

Recently, VTA GABA neurons were shown to encode reward expectation with a persistent cue response proportional to the expected reward (Cohen et al., 2012; Eshel et al., 2015; Tian et al., 2016). Additionally, selectively exciting and inhibiting VTA GABA neurons during a classical-conditioning task, Eshel et al. (2015) revealed that these neurons are likely source of the substraction operation, contributing to the inhibitory expectation signal in the RPE computation by DA neurons.

Furthermore, the presence of nicotinic acetylcholine receptors (nAChRs) in the VTA (Pontieri et al., 1996; Maskos et al., 2005; Changeux, 2010; Faure et al., 2014) provides a potential common route for acetylcholine (ACh) and nicotine (Nic) in modulating dopamine activity during a Pavlovian-conditioning task.

Particularly, the high-affinity α4β2 subunit-containing nAChRs desensitizing relatively slowly (≃ sec) and located post-synaptically on VTA DA and GABA neurons have been shown to have the most prominent role in nicotine-induced DAergic bursting activity and self-administration, as suggested by mouse knock-out experiments (Maskos et al., 2005; Changeux, 2010; Faure et al., 2014) and recent direct optogenetic modulation of these somatic receptors (Durand-de Cuttoli et al., 2018).

We have previously developed and validated a population level circuit dynamics model (Graupner et al., 2013; Tolu et al., 2013; Maex et al., 2014; Dumont et al., 2018) of the influence nicotine and Ach interplay may have on the VTA dopamine cell activity. Using this model we showed that Nic action on α4β2 could result in either direct stimulation or disinhibition of DA neurons. The latter scenario suggests that relatively low nicotine concentrations (~500 nM) during and after smoking preferentially desensitize α4β2 nAChRs on GABA neurons (Fiorillo et al., 2008). The endogenous cholinergic drive to GABA neurons would then decrease, resulting in decreased GABA neurons activity, and finally a disinhibition of DA neurons as confirmed *in vitro* (Mansvelder et al., 2002) and suggested by Graupner et al. (2013), Tolu et al. (2013), Maex et al. (2014), and Dumont et al. (2018) modeling work. Interestingly, this scenario requires that the high affinity nAChRs are in a pre-activated state, so that nicotine can desensitize them, which in turn implies a sufficiently high ambient cholinergic tone in the VTA. However, when the ACh tone is not sufficient, in this GABA-nAChR scenario, nicotine would lead to a significant inhibition of the DA neurons. Furthermore, a recent study showed that optogenetic inhibition of PPTg cholinergic fibers inhibit only the VTA non-DA neurons (Yau et al., 2016), suggesting that ACh acts preferentially on VTA GABA neurons. However, the effects of Nic and ACh on dopamine responses to rewards via α4β2-nAChRs desensitization during classical-conditioning have remained elusive.

In addition to the above issues, a non-trivial issue arises from the timing structure of the conditioning tasks. Typically, the reward to be consumed is delivered after a temporal delay past the conditioning cue, which begs important related questions: how is the reward information transferred from the reward-delivery time to the earlier reward-predictive stimulus and how does the brain compute the precise timing of reward? In other words, how is the relative co-timing of the reward and the reinforcer learned in the brain? These issues generate further lines of enquiry on how this learning process may be altered by nicotine. In order to start clarifying the possible neural mechanisms underlying the observed RPE-like activity in DA neurons, we propose here a simple neuro-computational model inspired from Graupner et al. (2013), incorporating the mean dynamics of four neuron populations: the prefrontal cortex (PFC), the pedunculopontine tegmental nucleus (PPTg), the VTA dopamine and GABA neurons.

Note that we explicitly choose to base our model on the desensitization scenario from Graupner et al. (2013), where the nicotinic receptors are relatively efficient in controlling the GABA neuron populations activity. In this case, the positive dopamine response to nicotine is due to α4β2-nAChRs desensitization and requires a relatively high endogenous cholinergic tone-for low acetylcholine tone, nicotine is predicted to depress DA output in this scheme. Since the animal is performing experimental tasks in a state of cognitive effort, the disinhibition scenario we surmise could be relevant as it implies a high cholinergic tone impinging onto the VTA (Picciotto et al., 2008, 2012).

Taking into account recent neurobiological data, particularly showing the activity of VTA GABA neurons during classical-conditioning (Cohen et al., 2012; Eshel et al., 2015), we qualitatively and quantitively reproduce several aspects of a Pavlovian-conditioning task—which we take as a paradigmatic example of reward-based conditioning—such as the phasic components of dopaminergic activation with respect to reward magnitude, omission and timing, the working-memory activity in the PFC, the response of the PPTg to primary rewards, and the dopamine-induced plasticity in cortical and corticostriatal synapses.

Having built the minimal model that incorporates the influence of nAChRs on the computations of reward-related learning signals in the VTA circuit, we are poised to use the model to examine how acute nicotine may affect this computation. Notably, we qualitatively assessed the potential effects of nicotine-induced desensitization of α4β2-nAChRs on GABA neurons, leading to a disinhibition of DA burst-response to rewarding events. As we will show below, this effect would lead to pathological changes in evaluation of rewards and stimuli associated with nicotine and lead to a bias in boosting strong vs. weak rewards as observed recently experimentally. These last simulations imply an important role for nicotine in not only provoking a positive over-valuation of acute nicotine itself, but also in having an impact on the general rewarding quality of nicotine-associated environments. Additionally, our simulations also imply a heightened reward sensitivity in animals exposed to nicotine. We further analyze the potential behavioral and motivational implications of these predicted effects in the Discussion section.

## 2. Methods: Computational Model and Simulated Behavioral Tasks

**Figure 1:**
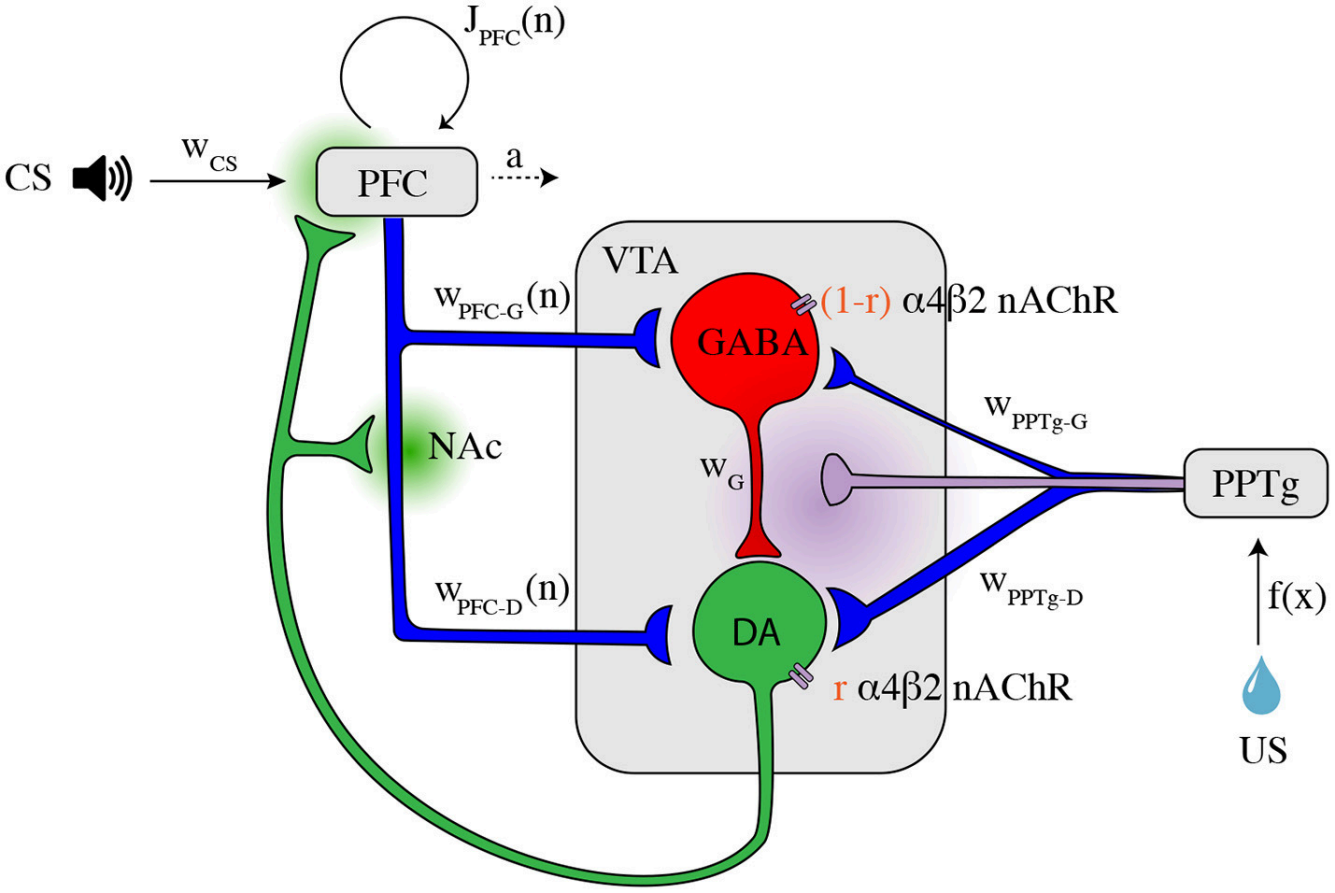
Illustration of the VTA circuit and neural dynamics of each area during learning of a pavlovian-conditioning task. Afferents inputs and circuitry of the ventral tegmental area (VTA). The GABA neuron population (red) inhibits locally the DA neuron population (green). This local circuit receives excitatory glutamatergic input (blue axons) from the corticostriatal pathway and the pedunculopontine tegmental nucleus (PPTg). The PPTg furthermore furnishes cholinergic projections (purple axon) to the VTA neurons (α4β2 nAChRs). *r* is the parameter to change continuously the dominant site of α4β2 nAChR action. Dopaminergic efferents (green axon) project, amongst others, to the nucleus accumbens (NAc) and the prefrontal cortex (PFC) and modulates cortico-striatal projections *w*_PFC-D_ and *w*_PFC-G_ and PFC recurrent excitation *J*_PFC_ weights. The PFC integrates CS (tone) information, while the PPTg respond phasically to the water reward itself (US). Dopamine and acetylcholine outflows are represented by green and purple shaded areas, respectively. All parameters and description are summarized in Supplementary Table 1.

In order to examine the VTA circuit level mechanisms of reward prediction error computation and effects of nicotine on this activity during classical-conditioning, we built a neural population model of the VTA and its afferent inputs inspired from the mean-field approach of Graupner et al. (2013). This model incorporates the DA and GABA neuronal populations in the VTA and their glutamatergic and cholinergic afferents from the PFC and the PPTg (Figure 1). Based on recent neurobiological data, we propose a model for the activity of the PFC and PPTg inputs during classical-conditioning contributing to the observed VTA GABA and DA activity. Additionally, the activation and desensitization dynamics of the nAChR-mediated currents in response to Nic and ACh were described by a 4-state model taken from Graupner et al. (2013).

### 2.1. Mean-Field Description of VTA Neurons and Their Afferents

First, the model from Graupner et al. (2013) describing the dynamics of VTA neuron populations and the effects of Nic and ACh on nAChRs was re-implemented with several quantitative modifications according to experimental data.

The temporal dynamics of the average activities of DA and GABA neurons in the VTA taken from Graupner et al. (2013) are described by the following equations:

(1) $$\begin{array}{l} \left\{ \begin{array}{l} \tau_{\text{D}}\frac{d\nu_{\text{D}}}{dt}=-\nu_{\text{D}}+F(B_{\text{D}}-I_{\text{G-D}}+I_{\text{Glu-D}}+rI_{\alpha 4\beta 2})\\ \tau_{\text{G}}\frac{d\nu_{\text{G}}}{dt}=-\nu_{\text{G}}+\Phi (B_{\text{G}}+I_{\text{Glu-G}}+(1-r)I_{\alpha 4\beta 2}), \end{array}\right. \end{array}$$

where ν_D_ and ν_G_ are the mean firing rates of the DA and GABAergic neuron populations, respectively. τ_D_ = 30 ms and τ_G_ = 30 ms are the membrane time constants of both neuron populations specifying how quickly the neurons integrate input changes. *I*_Glu_ characterize the excitatory inputs from PFC and PPTg mediated by glutamate receptors. *I*_α4β2_ represent the excitatory input mediated by α4β2-containing nAChRs, activated by PPTg ACh input and Nic. *I*_G-D_ is the local feed-forward inhibitory input to DA neurons emanating from VTA GABA neurons. *B*_D_ = 18 and *B*_G_ = 14 are the baseline firing rates of each neuron population in the absence of external inputs, according to Eshel et al. (2015) experimental data - with external inputs, the baseline activity of DA neurons is around 5 Hz.

The parameter *r* sets the balance of α4β2 nAChR action through GABA or DA neurons in the VTA. For *r* = 0, they act through GABA neurons only, whereas for *r* = 1 they influence DA neurons only. Φ(.) is the linear rectifier function, which only keeps the positive part of the operand and outputs 0 when it is negative. *F*(.) is a non-linear sigmoid transfer function for the dopaminergic neurons enabling to describe the high firing rates in the bursting mode and the low frequency activity in the tonic (pacemaker) mode, and their slow variation below their baseline activity with external inputs (≃ 5 Hz):

(2) $$\begin{array}{l} F(x)=\frac{\omega }{1+\exp(-\beta (x-\gamma ))}, \end{array}$$

where ω = 30 represent the maximum firing rate, γ = 8 is the inflection point and β = 0.3 is the slope. These parameters were chosen in order to account for bursting activity of DA neurons starting from a certain threshold (γ) of input and their maximal activity observed *in vivo* (Hyland et al., 2002; Eshel et al., 2015). Indeed, physiologically, high firing rates (>8 Hz) are only attained during DA bursting activity and not tonic activity (≃ 5 Hz).

The input currents in Equation (1) are given by:

(3) $$\begin{array}{l} \left\{ \begin{array}{l} I_{\text{G-D}}(t)=w_{\text{G-D}}\cdot \nu_{\text{G}}(t)\\ I_{\text{Glu-D}}(t)=w_{\text{PFC-D}}(n)\cdot \nu_{\text{PFC}}(t)+w_{\text{PPT-D}}\cdot \nu_{\text{PPT}}(t)\\ I_{\text{Glu-G}}(t)=w_{\text{PFC-G}}(n)\cdot \nu_{\text{PFC}}(t)+w_{\text{PPT-G}}\cdot \nu_{\text{PPT}}(t)\\ I_{\alpha 4\beta 2}(t)=w_{\alpha 4\beta 2}\cdot v_{\alpha 4\beta 2}(t), \end{array}\right. \end{array}$$

where *w*_x_'s (with x = G-D, PFC-D, PFC-G, PPT-D, PPT-G, α4β2) specify the total strength of the respective input (Figure 1 and Supplementary Table 1). For instance, *w*_PPT-D_ specifies the strength of the connection from the PPTg to the DA population.

The weight of α4β2-nAChRs, *w*_α4β2_ = 15 was chosen in order to account for the increase of baseline firing rates compared to Graupner et al. (2013) where *w*_α4β2_ = 1, *B*_D_ = 0.1 and *B*_G_ = 0. We also assumed that the PFC-DA and PFC-GABA connections were equal, which leads to the following important equality: *w*_PFC-D_(*n*) = *w*_PFC-G_(*n*) for any trial *n*.

In summary, inhibitory input to DA cells, *I*_G-D_, depends on GABA neuron population activity, ν_G_ (Eshel et al., 2015). Excitatory input to DA and GABA cells depends on PFC-NAc (Ishikawa et al., 2008; Keiflin and Janak, 2015) and PPTg (Lokwan et al., 1999; Yoo et al., 2017) glutamatergic inputs activities, ν_PFC_ and ν_PPT_ respectively (see next section). The activation of α4β2 nAChRs, ν_α4β2_, determines the level of direct excitatory input *I*_α4β2_ evoked by nicotine or acetylcholine (see last section).

### 2.2. Neuronal Activities During Classical-Conditioning

As described above, previous studies identified signals from distinct brain areas that could be responsible for VTA DA neuron activity during classical conditioning. We thus consider a simple model that particularly accounts for Eshel et al. (2015) experimental data on VTA GABA neurons activity. In this approach, we propose that the sustained activity reflecting reward expectation in GABA neurons comes from the PFC (Schoenbaum et al., 1998; Le Merre et al., 2018), that sends projections on both VTA DA and GABA neurons through the NAc (Morita et al., 2013; Keiflin and Janak, 2015). The PFC-NAc pathway thus drives feed-forward inhibition onto DA neurons by exciting VTA GABA neurons that in turn inhibit DA neurons (Figure 1). Second, we consider that a subpopulation of the PPTg provides the reward signal to the dopamine neurons at the US (Kobayashi and Okada, 2007; Okada et al., 2009).

#### 2.2.1. Classical-Conditioning Task and the Associated Signals

**Figure 2:**
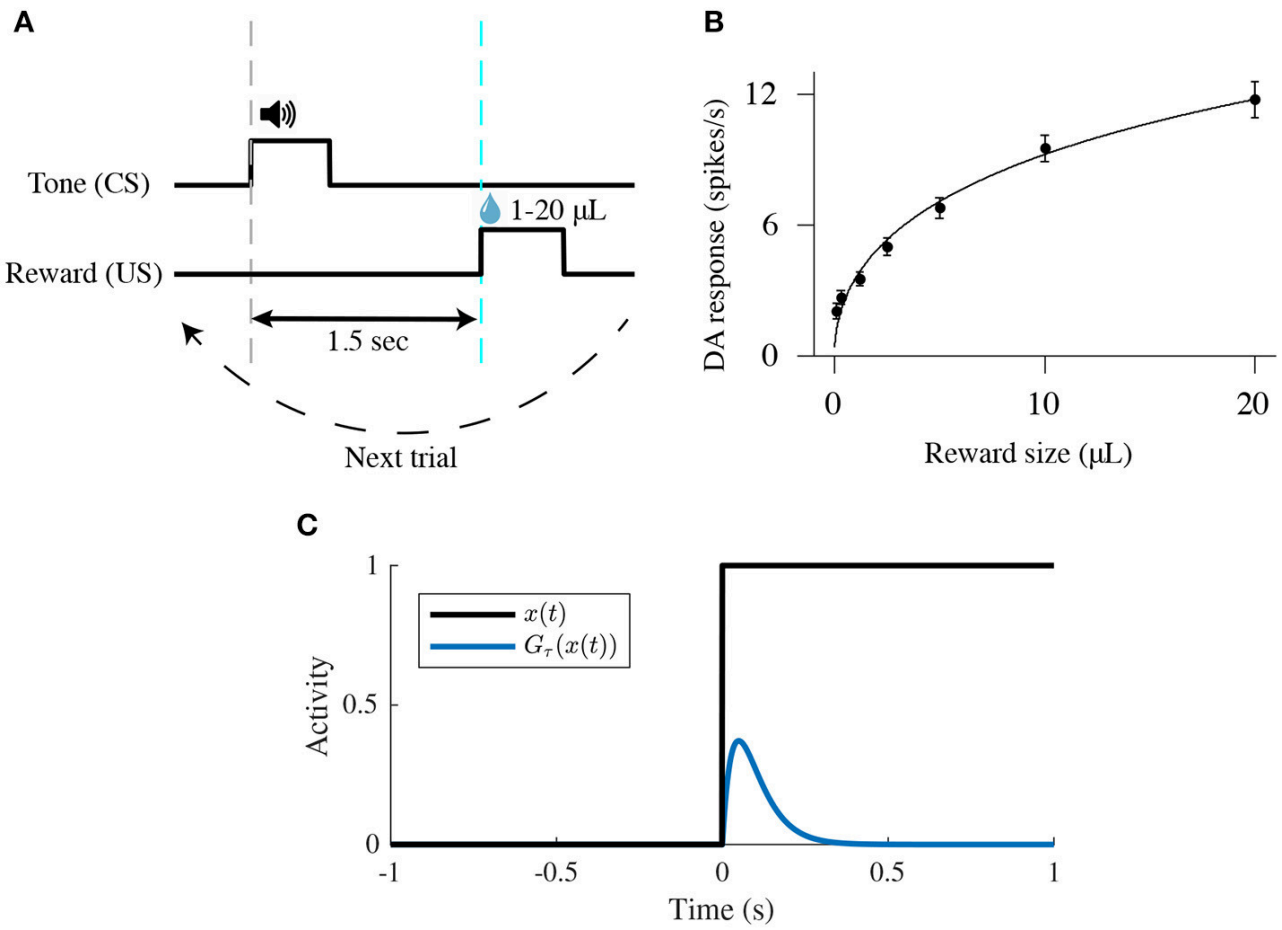
Schematic of a classical-conditioning task. **(A)** Simulated thirsty mice receive a water reward ranging from 1 to 20 μL. Tone (CS) and reward (US) onsets are separated by 1.5 s. **(B)** Firing rates [mean ± standard-error (s.e.)] of optogenetically identified dopamine neurons in response to different sizes of unexpected reward. Adapted from Eshel et al. (2016). **(C)** Temporal profile of the phasic function *G*_τ_(*x*(*t*)) (Equation 4) in response to a square input *x*(*t*).

We modeled a VTA neural circuit (Figure 1) while mice are classically conditioned with a tone stimulus that predicts an appetitive outcome as in Eshel et al. (2015), but with 100% probability. Each simulated behavioral trial begins with a conditioned stimulus (CS; a tone, 0.5 s), followed by an unconditioned stimulus (US; the outcome, 0.5 s) separated by an interval of 1.5 s. (Figure 2A). This type of task, implying a delay between the CS offset and the US onset (here, 1 s), is then a trace-conditioning task, that differs from a delay-conditioning task where the CS and US overlap (Connor and Gould, 2016).

As the animal learns that a fixed reward predictably follows a predictive tone at a specific timing, our model proposes possible underlying biological mechanisms of Pavlovian-conditioning in PPTg, PFC, VTA DA, and GABA neurons (Figure 1).

As represented in previous models (O'Reilly et al., 2007; Vitay and Hamker, 2014), the CS signal is modeled by a square function (ν_CS_(*t*)) equal to 1 during the CS presentation (0.5 s) and to 0 otherwise (Figure 2A). The US signal is modeled by a similar square function (ν_US_(*t*)) as the CS but is equal to the reward size during the US presentation (0.5 s) and 0 otherwise (Figure 2A).

#### 2.2.2. Neural Representation of the US Signal in the PPTg

Dopamine neurons in the VTA exhibit a relatively low tonic activity (around 5 Hz), but respond phasically with a short-latency (< 100 ms), short-duration (< 200 ms) burst of activity in response to unpredicted rewards (Schultz, 1998; Eshel et al., 2015). These phasic bursts of activity are dependent on glutamatergic activation by a subpopulation of PPTg (Okada et al., 2009; Keiflin and Janak, 2015; Yoo et al., 2017) found to discharge phasically at reward delivery, with the levels of activity associated with the actual reward and not affected by reward expectation.

To integrate the US input into a short-term phasic component we use the function *G*_τ_(*x*(*t*)) (Vitay and Hamker, 2014) defined as follows:

(4) $$\begin{array}{l} \left\{ \begin{array}{l} \tau {\dot{x}}_{1}(t)=-x_{1}(t)+x(t)\\ \tau {\dot{x}}_{2}(t)=-x_{2}(t)+x_{1}(t)\\ G_{\tau }(x(t))=\Phi (x_{1}(t)-x_{2}(t)). \end{array}\right. \end{array}$$

Here when *x*(*t*) switches from 0 to 1 at time *t* = 0, *G*_τ_(*x*(*t*)) will display a localized bump of activation with a maximum at *t* = τ. This function is thus convenient to integrate the square signal ν_US_(*t*) into a short-latency response (Figure 2C).

Furthermore, dopamine response amplitudes to unexpected rewards follow a simple saturating function (fitted by a Hill function in Figure 2B) (Eshel et al., 2015, 2016). We thus consider that PPTg neurons respond to the reward delivery signal (US) in a same manner as DA neurons i.e., with a saturating dose-response function:

(5) $$\begin{array}{l} \left\{ \begin{array}{l} \nu_{\text{PPTg}}(t)=G_{\tau_{\text{PPTg}}}[f(\nu_{\text{US}}(t))]\\ f(x)=f_{\text{max}}(\frac{x^{0.5}}{x^{0.5}+h^{0.5}}), \end{array}\right. \end{array}$$

where ν_PPTg_ is the mean activity of the PPTg neurons population, τ_PPTg_ = 100 ms (the short-latency response), and *f*(*x*) is a Hill function with two parameters: *f*_max_, the saturating firing rate; and *h*, the reward size that elicits half-maximum firing rate. Here, we chose *f*_max_ = 70 and *h* = 20 in order to obtain a similar dose-response curve once PPTg activity is transferred to DA neurons as in Eshel et al. (2016) (Figure 2B).

#### 2.2.3. Neural Representation of CS Signal in the PFC

In addition to their response to unpredicted rewards, learning drives the DA neurons to respond to reward-predictive cues and to reduce their response at the US (Schultz et al., 1997; Schultz, 1998; Matsumoto and Hikosaka, 2009; Eshel et al., 2015). Neurons in the PFC respond to these cues through a sustained activation starting at the CS onset and ending at the reward-delivery (Connor and Gould, 2016; Le Merre et al., 2018). Furthermore, this activity has been shown to increase in the early stage of a classical-conditioning learning task (Schoenbaum et al., 1998; Le Merre et al., 2018). Especially, the PFC participates in the association of temporally separated events in trace-conditioning task through working-memory mechanisms (Connor and Gould, 2016), maintaining a representation of the CS accross the CS-US interval, and this timing-association is dependent on dopamine modulation in the PFC (Puig et al., 2014; Popescu et al., 2016).

We thus assume that the PFC integrates the CS signal and learns to maintain its activity until the reward delivery. Consistently with previous neural-circuit working-memory models (Durstewitz et al., 2000), we minimally described this mechanism by a neural population with recurrent excitation and slower adaptation dynamics blue (e.g., increase in calcium-dependent potassium hyperpolarizing currents *I*_KCa_) inspired from Gerstner et al. (2014):

$$\begin{array}{l} \left\{ \begin{array}{l} \tau_{\text{PFC}}\frac{d\nu_{\text{PFC}}}{dt}=-\nu_{\text{PFC}}(t)+F[w_{\text{CS}}\cdot \nu_{\text{CS}}(t)\\ \;+J_{\text{PFC}}(n)\cdot \nu_{\text{PFC}}(t)-a(t)]\\ \tau_{a}\frac{da}{dt}=a_{\infty }(\nu_{\text{PFC}})-a(t), \end{array}\right. \end{array}$$

where τ_PFC_ = 100 ms (short-latency response), *a*(*t*) describes the amount of adaptation that neurons have accumulated, *a*_∞_ = *c*·ν_PFC_ is the asymptotic level of adaptation that is attained by a slow time constant τ_*a*_ = 1, 000 ms (Gerstner et al., 2014) if the population continuously fires at a contant rate ν_PFC_, *J*_PFC_(*n*) represents the strength of the recurrent excitation exerted by the PFC depending on the learning trial *n* (initially *J*(1) = 0.2), *w*_CS_ the strength of the CS input. *F*(*x*) is the non-linear sigmoid transfer function defined in Equation (2) allowing the emergence of bistability network. We chose ω = 30, γ = 8 and β = 0.5 in order to account for the PFC activity changes in working-memory tasks (Connor and Gould, 2016).

#### 2.2.4. Learning of the US Timing in the PFC

The dynamical system described above typically switches between two stables states: quasi absence of activity or maximal activity in the PFC. The latter stable state particularly appears as *J*_PFC_(*n*) increases with learning:

(6) $$\begin{array}{l} J_{\text{PFC}}(n+1)\leftarrow J_{\text{PFC}}(n)+\alpha_{T}\cdot \Delta t_{\text{DA}}, \end{array}$$

where α_*T*_ = 0.2 is the timing learning rate, Δ*t*_DA_ = *t*_2_ − *t*_1_ measures the difference between the time at which PFC activity declines (*t*_1_ such as ν_PFC_(*t*_1_) ≃ γ after CS onset) and the time of DA maximal activity at the US, *t*_2_. This learning mechanism of reward timing, simplified from Luzardo et al. (2013), triggers the increase of the recurrent connections (*J*_PFC_) through dopamine-mediated modulation in the PFC (Puig et al., 2014; Popescu et al., 2016) such as ν_PFC_ collapses at the time of reward delivery. This learning process occurs in the early stage of the task (Le Merre et al., 2018) and is therefore much faster than the learning of reward expectation.

#### 2.2.5. Learning of Reward Expectation in Cortico-Striatal Connections

According to studies showing a DA-dependent cortico-striatal plasticity (Reynolds et al., 2001; Yagishita et al., 2014; Keiflin and Janak, 2015), we assumed that the reward value predicted from the tone (CS) is stored in the strength of cortico-striatal connections [*w*_PFC-D_(*n*) and *w*_PFC-G_(*n*)], i.e., between the PFC and the NAc, and is updated through plasticity mechanisms depending on phasic dopamine response after reward delivery as in the following equation proposed by Morita et al. (2013):

(7) $$\begin{array}{l} \left\{ \begin{array}{l} w_{\text{PFC-D}}(n+1)\leftarrow w_{\text{PFC-D}}(n)+\alpha_{\text{V}}\cdot \delta (n)\\ w_{\text{PFC-G}}(n+1)\leftarrow w_{\text{PFC-G}}(n)+\alpha_{\text{V}}\cdot \delta (n), \end{array}\right. \end{array}$$

where α_V_ is the cortico-striatal plasticity learning rate related to reward magnitude, δ(*n*) is a deviation from the DA baseline firing rate, computed by the area under curve of ν_D_ in a 200 ms time-window following US onset, above a baseline defined by the value of ν_D_ at the time of US onset. δ(*n*) is thus the reward-prediction error signal that updates the reward-expectation signal stored in the strength of the PFC input *w*_PFC-D_(*n*) until the value of the reward is learned (Rescorla and Wagner, 1972).

This assumption was taken from Morita et al. (2013) modeling work and various hypotheses on dopamine-mediated plasticity in associative-learning (Keiflin and Janak, 2015) and recent experimental data (Yagishita et al., 2014; Fisher et al., 2017). It implies that the excitatory signal from the PFC first activates the nucleus accumbens (NAc) and is then transferred via the direct disinhibitory pathway to the VTA. Here, we then considered that *w*_PFC-D_ and *w*_PFC-G_ are provided by the PFC-NAc pathway but we did not explicitly represent the NAc population (Figure 1).

#### 2.2.6. Cholinergic Input Activity

Our model also reflects the cholinergic (ACh) afferents to the DA and GABA cells in the VTA (Dautan et al., 2016; Yau et al., 2016). The α4β2 nAChRs are placed somatically on both the DA and the GABA neurons and their activity depends on ACh and Nic concentration within the VTA (see last section). As PPTg was found to be the main source of cholinergic input to the VTA, we assume that ACh concentration directly depends on PPTg activity, as modeled by the following equation:

(8) $$\begin{array}{l} ACh(t)=w_{\text{ACh}}\cdot \nu_{\text{PPTg}}(t), \end{array}$$

where *w*_ACh_ = 1 μM is the amplitude of the cholinergic connection that tunes concentration of acetylcholine *ACh* (in μM) at a physiologically relevant concentration (Graupner et al., 2013).

### 2.3. Modeling the Activation and Desensitization of nAChRs

We implemented nAChR activation and desensitization from Graupner et al. (2013) as transitions of two independent state variables: an activation gate and a desensitization gate. The nAChR receptors can then be in four different states: deactivated/sensitized, activated/sensitized, activated/desensitized and deactivated/desensitized. The receptors are activated in response to both Nic and ACh, while desensitization is driven by Nic only (if η = 0). Once Nic or ACh is removed, the receptors can switch from activated to deactivated and from desensitized to sensitized.

The mean total activation level of nAChRs (ν_α4β2_) is modeled as the product of the activation rate *a* (fraction of receptors in the activated state) and the sensitization rate *s* (fraction of receptors in the sensitized state). The total normalized nAChR activation is therefore: ν_α4β2_ = *a*·*s*. The time course of the activation and the sensitization variables is given by:

(9) $$\begin{array}{l} \frac{dy}{dt}=\frac{y_{\infty }(Nic,ACh)-y}{\tau_{y}(Nic,ACh)}, \end{array}$$

where τ_*y*_(*Nic, ACh*) refers to the Nic/ACh concentration-dependent time constant at which the steady-state *y*_∞_(*Nic, ACh*) is achieved. The maximal achievable activation or sensitization, for a given Nic/ACh concentration, *a*_∞_(*Nic, ACh*) and *s*_∞_(*Nic, ACh*) are given by Hill equations of the form:

(10) $$\begin{array}{l} \left\{ \begin{array}{l} a_{\infty }(Nic,ACh)=\frac{{\left(ACh+\alpha Nic\right)}^{n_{\text{a}}}}{EC_{50}{\;}^{n_{\text{a}}}+{\left(ACh+\alpha Nic\right)}^{n_{\text{a}}}}\\ s_{\infty }(Nic,ACh)=\frac{IC_{50}{\;}^{n_{\text{s}}}}{IC_{50}{\;}^{n_{\text{s}}}+{\left(Nic+\eta ACh\right)}^{n_{\text{s}}}}, \end{array}\right. \end{array}$$

where *EC*_50_ and *IC*_50_ are the half-maximal concentrations of nAChR activation and sensitization, respectively. The factor α>1 accounts for the higher potency of Nic to evoke a response as compared to ACh: α_α4β2_ = 3. *n*_a_ and *n*_s_ are the Hill coefficients of activation and sensitization. η varies between 0 and 1 and controls the fraction of the ACh concentration driving receptor desensitization. Here, as we only consider Nic-induced desensitization, we set η = 0.

As the transition from the deactivated to the activated state is fast (~μs), the activation time constant τ_*a*_ was simplified to be independent on ACh and Nic concentration: τ_*a*_(*Nic, ACh*) = τ_*a*_ = *const*. The time course of Nic-driven desensitization is characterized by a concentration-dependent time constant

(11) $$\begin{array}{l} \tau_{d}(Nic,ACh)=\tau_{0}+\tau_{\text{max}}\frac{K_{\tau }{\;}^{n_{\tau }}}{K_{\tau }{\;}^{n_{\tau }}+{\left(Nic+\eta ACh\right)}^{n_{\tau }}}, \end{array}$$

where τ_max_ refers to the recovery time constant from desensitization in the absence of ligands, τ_0_ is the fastest time constant at which the receptor is driven into the desensitized state at high ligand concentrations. *K*_τ_ is the concentration at which the desensitization time constant attains half of its minimum. All model assumptions are further described in Graupner et al. (2013).

### 2.4. Simulated Experiments

#### 2.4.1. Optogenetic Inhibition of VTA GABA Neurons

In order to qualitatively reproduce (Eshel et al., 2015) experimental data, we simulated the photo-inhibition effect in a subpopulation of VTA GABA neurons with an exponential decrease between *t* = 1.5 s and *t* = 2.5s (±500 ms around reward-delivery). First, the light was modeled by a square signal ν_light_ equal to the laser intensity *I* = 4 for 1.5 < *t* < 2.5 and zero otherwise. Then, we subtracted this signal to VTA GABA neuron activity as follows:

(12) $$\begin{array}{l} \left\{ \begin{array}{l} \tau_{\text{s}}\frac{ds}{dt}=-s(t)+\nu_{\text{light}}(t)\\ \nu_{\text{G-opto}}=\nu_{\text{G-control}}-s(t), \end{array}\right. \end{array}$$

where *s* is the subtracted signal that integrates the light signal ν_light_ with a time constant τ_s_ = 300 ms, ν_G-opto_ is the photo-inhibited GABA neurons activity, and ν_G-control_ is the normal GABA neurons activity with no opto-inhibition. All parameters (*I*, τ_s_) were chosen in order to reproduce qualitatively the photo-inhibition effects revealed by Eshel et al. (2015) experiments. Furthermore, as the effects of GABA photo-inhibition onto DA neurons appear to be relatively weak in Figure 3 of Eshel et al. (2015), we assumed that only a subpopulation of the total GABA neurons are photo-inhibited and we therefore applied (Equation 12) for only 20% of the VTA GABA population. This assumption was based on the partial expression of Archeorhodopsin (ArchT) in GABA neurons (Eshel et al., 2015, Extended Data Figure 1) and the other possible optogenetic effects (recording distance, variability of the response among the population, laser intensity, etc.).

#### 2.4.2. Nicotine Injection in the VTA

In order to model chronic nicotine injection in the VTA while mice perform classical-conditioning tasks with water reward, the above equations were simulated but after 5 min of 1 μ M Nic injection in the model for each trial. This process allowed to focus only on the effects of α4β2-nAChRs desensitization (see next section) during conditioning trials.

#### 2.4.3. Decision-Making Task

We simulated a protocol designed by Naudé et al. (2016) recording simultaneously the sequential choices of a mouse between three differently rewarding locations (associated with reward size) in a circular open-field (**Figure 7A**). These three locations form an equilateral triangle and provide respectively 2, 4, 8 μ L water rewards. Each time the mouse reaches one of the rewarding locations, the reward is delivered. However, the mouse receives the reward only when it alternates between rewarding locations.

Before the simulated task, we considered that the mouse has already learned the value of each location (pre-training) and thus knows the expected associated reward. Each value was computed taking the maximal activity of DA neurons within a time window following the CS onset (here, the view of the location) for the three different reward sizes after learning. We also considered that each time the mouse reaches a new location, it enters in a new state *i*. Decision making-models inspired from Naudé et al. (2016) determine the probability *P*_*i*_ of choosing the next state *i* as a function of the expected value of this state. Because mice could not return to the same rewarding location, they had to choose between the two remaining locations. We thus modeled decisions between two alternatives. The probability *P*_*i*_ was computed according to the softmax choice rule:

(13) $$\begin{array}{l} P_{i}=\frac{1}{\exp(b(V_{j}-V_{i})}, \end{array}$$

where *V*_*i*_ and *V*_*j*_ are the values of the states *i* and *j* (the other option), respectively, *b* is an inverse temperature parameter reflecting the sensitivity of choice to the difference between both values. We chose *b* = 0.4 which corresponds to a reasonable exploration-exploitation ratio.

We simulated the task over 10,000 simulations and computed the number of times the mouse chose each location. We thus obtained the average repartition of the mouse over the three locations. A similar task was simulated for mice after 5 min Nic ingestion (see below).

## 3. Results

We used the model developed above to understand the learning dynamics within the PFC-VTA circuitry and the mechanisms by which the RPE in the VTA is constructed. Our minimal circuit dynamics model of the VTA was inspired from Graupner et al. (2013) and modified according to recent neurobiological studies (see Methods) in order to reproduce RPE computations in the VTA. This model reflects the glutamatergic (from PFC and PPTg) and cholinergic (from PPTg) afferents to VTA DA and GABA neurons, as well as local inhibition of DA neurons by GABA neurons. We also included the activation and desensitization dynamics of α4β2 nAChRs from Graupner et al. (2013), placed somatically on both DA and GABA neurons, depending on a fraction parameter *r*.

We note that we explicitly set *r* so the majority of nAChRs are located on the inhibitory GABA interneurons, hence following the “disinhibition” scheme as per (Graupner et al., 2013).

We simulated the proposed PFC and PPTg activity during the task, where corticostriatal connections between the PFC and the VTA and recurrent connections among the PFC were gradually modified by dopamine in the NAc. Finally, we studied the potential influence of nicotine exposure on DA responses to rewarding events.

**Figure 3:**
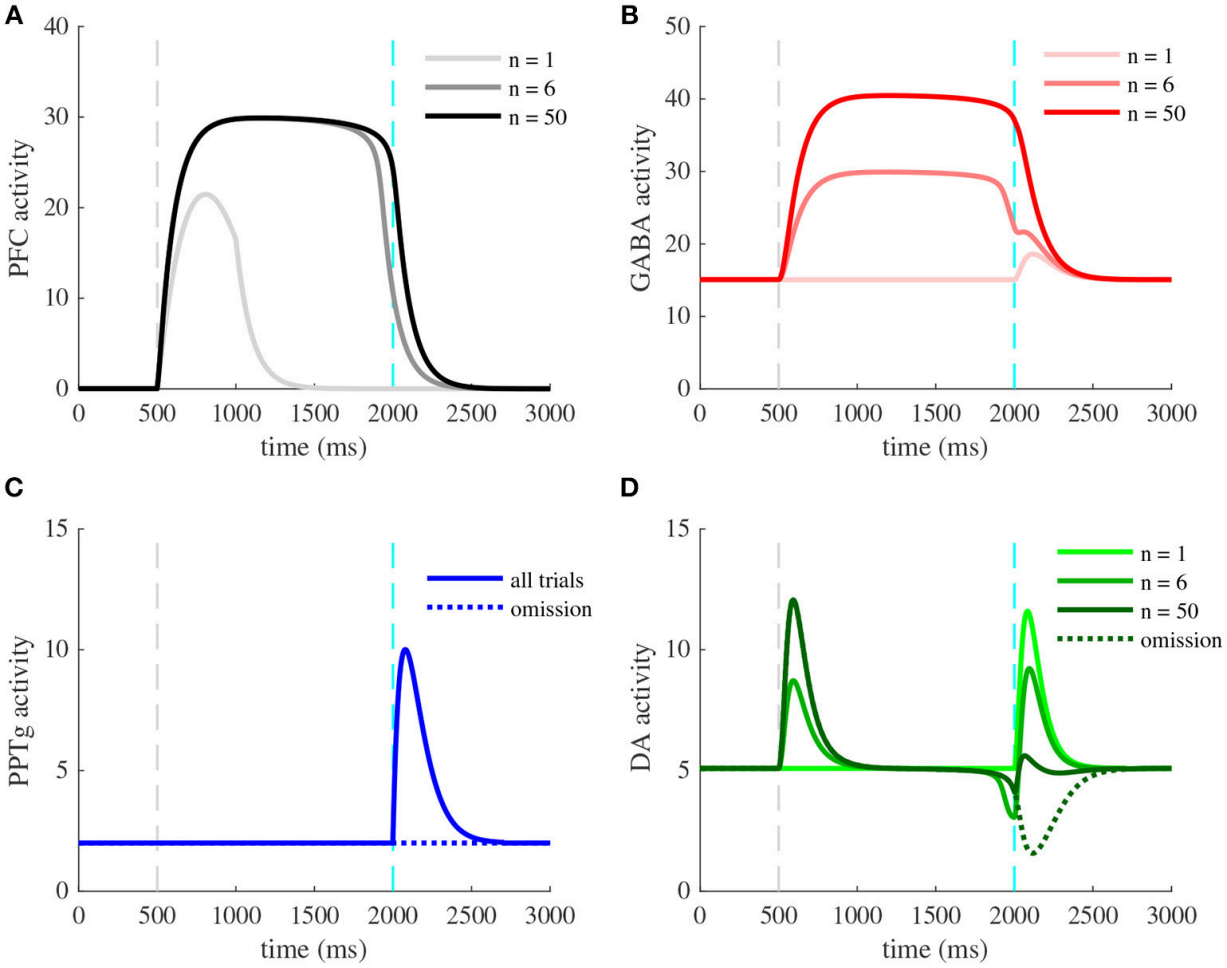
Activity of VTA neurons and their afferents during a pavlovian-conditioning task. Simulated mean activity (Hz) of each neuron population during a pavlovian-conditioning task, where a tone is presented systematically 1.5 s before a water reward (4 μL). Three different trials are represented: the initial conditioning trial (*n* = 1, light colors), an intermediate trial (*n* = 6, medium colors) and the final trial (*n* = 50, dark colors) and when reward is omitted after learning (dotted lines). Vertical dashed gray and cyan lines represent CS and US onsets, respectively. **(A)** PFC neurons learn the timing of the task by maintaining their activity until US. **(C)** PPTg neurons activity responds to the US signal at all trials. **(B)** VTA GABA persistent activity increases with learning, **(D)** VTA DA activity increase at the CS and decrease at the US.

We should note that most experiments we simulated herein concern the learning task of a CS-US association (Figure 2). The learning procedure consists of a conditioning phase where a tone (CS) and a constant water-reward (US) are presented together for 50 trials. Within each 3 s-trial, the CS is presented at *t* = 0.5 s (Figures 3, **5**, **6**, dashed gray line) followed by the US at *t* = 2 s (Figures 3, **5**, **6**, dashed cyan line).

### 3.1. Pavlovian-Conditioning Task and VTA Activity

DA activity during a classical-conditioning task was first recorded by Schultz (1998) and tested in further several studies. Additionally, Eshel et al. (2015) also recorded the activity of their putative neighboring neurons, the VTA GABA neuron population. Our goal was first to qualitatively reproduce VTA GABA and DA activity during associative learning of a pavlovian-conditioning task.

In order to understand how different brain areas interact during the conditioning and also during reward omission, we examined the simulated time course of activity of four populations (PFC, PPTg, VTA DA and GABA), Figure 3, at the initial conditioning trial (*n* = 1, light color curves), an intermediary trial (*n* = 6, medium color curves) and at the final trial (*n* = 50, dark color curves). In line with experiments, the reward delivery (Figure 3, dashed cyan lines) activates the PPTg nucleus (Figure 3C) at each conditioning trial. These neurons activate in turn VTA DA and GABA neurons through glutamatergic connections, causing a phasic burst in DA neurons at the US when the reward is unexpected (Figure 3D, *n* = 1), and a small excitation in GABA neurons (Figure 3B, *n* = 1). PPTg fibers also stimulate VTA neurons through ACh-mediated α4β2 nAChRs activation, with a larger influence on GABA neurons (*r* = 0.2 in Figure 1).

**Figure 4:**
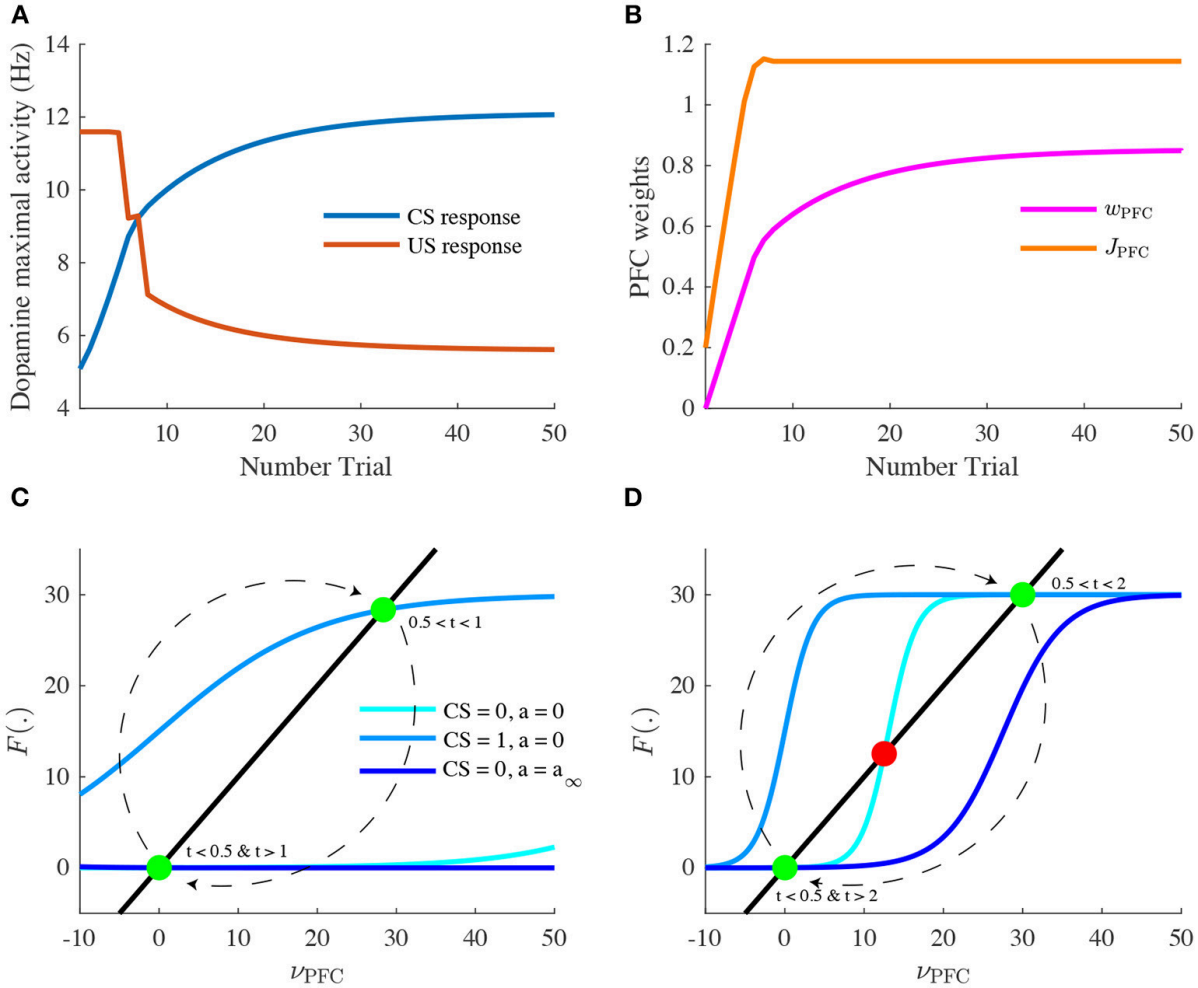
Learning of reward timing and magnitude during classical-conditioning. **(A)** The maximal activity of the VTA DA neurons at the CS onset (blue line) and at the reward delivery (orange line) is plotted for each trial of the conditioning task. These values are computed by taking the maximum value of the firing rate of the DA neurons in a small time window (200 ms) after the CS and the US onsets. **(B)** PFC weights showing two phases of learning: learning of the US timing by PFC recurrent connections weight (*J*_PFC_, orange line) and learning of the reward value by the weights of PFC neurons onto VTA neurons (*w*_PFC-D_ and *w*_PFC-G_, magenta line). **(C,D)** Phase analysis of PFC neuron activity from Equation (8) before learning **(C)** and after learning **(D)**. Different times of the task are represented: *t* < 0.5 s (before CS onset, light blue) and 1 s < *t* < 2 s (between CS offset and US onset, light blue), 0.5 s < *t* < 1 s (during CS presentation, medium blue) and *t* > 2 s (after US onset, dark blue). Fixed points are represented by green (stable) or red (unstable) dots. Dashed arrows: trajectories of the system from *t* = 0 to *t* = 3 s.

Early in the conditioning task, simulated PFC neurons respond to the tone (Figure 3A, *n* = 1), and this activity builds up until being maintained during the whole CS-US interval (Figure 3A, *n* = 6, *n* = 50). Thus, PFC neurons show a working-memory like activity now tuned to decay at the reward delivery time. Concurrently, the phasic activity of DA neurons at the US acts as prediction-error signal on corticostriatal synapses, increasing the glutamatergic input from the NAc onto VTA DA and GABA neurons (Figures 3B,D, 4B). Note that the NAc was not modeled explicitly, but we modeled the net effect of the PFC-NAc plasticity with the variables *w*_PFC-D_ and *w*_PFC-G_ (see next section).

Consequently, with learning, VTA GABA neurons show a sustained activation during the CS-US interval (Figure 3B, *n* = 6, *n* = 50) as found in Eshel et al. (2015) experiments and in turn inhibit their neighboring dopamine neurons. Thus, in DA neurons, the GABA neurons-induced inhibition occurs with a slight delay after the PFC-induced excitation, resulting in a phasic excitation at the CS and a phasic inhibition at the US (Figure 3D, *n* = 50).

The latter inhibition progressively cancels the reward-evoked excitation by the PPTg glutamatergic fibers in DA neurons. It also accounts for the pause in DA firing when reward is omitted after learning (Figures 3B,D, *n* = 50, dashed lines). In order to test whether this cancellation mode is robust to changes in GABA and PPTg time constants, we represented VTA GABA and DA neurons activity by varying τ_PPTg_ and τ_G_ (Figure S1). It results in slight variations of GABA and DA amplitudes, but their dynamics remain qualitatively robust. Together, these results propose a simple mechanism for RPE computation the VTA and its afferents.

Let us now take a closer look at the evolution of the phasic activity of DA neurons and their PFC-NAc afferents during the conditioning task. Figure 4A shows the evolution of CS- and US-mediated DA peaks over the 50 conditioning trials. Firstly, the US-related bursts (Figure 4A, red line) remain constant in the early trials until the timing is learnt by the PFC recurrent connections *J*_PFC_ (Figure 4B, orange line) following Equation (6). Secondly, US and CS (Figure 4A, blue line) responses respectively decrease and increase over all trials, following a slower learning process from cortico-striatal connections (Figure 4B, magenta line) described by Equation (7). This two-speed learning process enables to qualitatively reproduce the DA dynamics found experimentally, with almost no effect outside the CS and US time-windows (Figure 4D).

Particularly, the graphical analysis of the PFC system enables us to understand the timing learning mechanism. From Equation (6), we can see where the two functions ν_PFC_ → ν_PFC_ and ν_PFC_ → *F*[*w*_CS_ · ν_CS_(*t*) + *J*(*n*) · ν_PFC_(*t*) − *a*(*t*)] intersect each other (fixed points analysis) at four different timings during the simulation: before and after the CS presentation (ν_CS_ = 0, *a* = 0), during CS presentation (ν_CS_ = 1, *a* = 0) and after the reward is delivered (ν_CS_ = 0, *a* = *a*_∞_). Before learning, as *J*_PFC_ is weak (Figure 4C), the system starts at one fixed point (ν_PFC_ = 0), then jumps to another stable point during CS presentation (ν_PFC_≃30) and immediately goes back to the initial point (ν_PFC_ = 0) after CS presentation (*t* = 1 s) as shown in Figure 3A. After learning (Figure 4D), the system initially shows the same dynamics but when the CS is removed, the system is maintained at the second fixed point (30 Hz) until reward delivery (Figure 3A, *n* = 50) due to its bistability after CS presentation (cyan curve). Finally, with the adaptation dynamics, the PFC activity decays right after reward delivery (Figure 4D, dark blue). Indeed, through this timing learning mechanism, the strength of the recurrent connections maintains the Up state activity of the PFC exactly until the US timing (Equation 6). Together, these simulations show a two-speed learning process that enables VTA dopamine neurons to predict the value and the timing of the water reward from PFC plasticity mechanisms.

### 3.2. Photo-Inhibition of VTA GABA Neurons Modulates Prediction Errors

**Figure 5:**
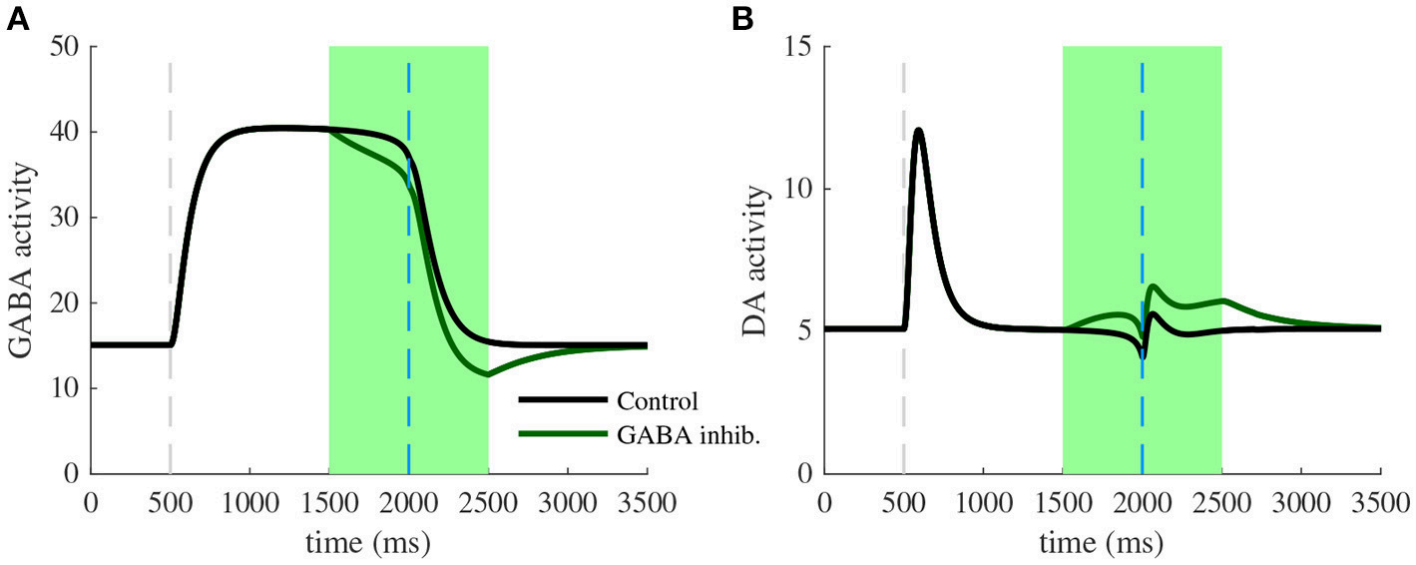
Photo-inhibition of VTA GABA neurons. **(A)** Activity of a subpopulation of GABA neurons (20%) in control (black) and with photo-inhibition (green) simulated by an exponential-like decrease of activity in a ±500 ms time-window around the US (green shaded area) after learning (*n* = 50). **(B)** DA activity resulting from GABA neurons activity in control condition (black) and when GABA is photo-inhibited (green) after learning (*n* = 50).

We next focus specifically on the local VTA neurons interactions at the end of the conditioning task. Particularly, we model the effects of VTA GABA optogenetic inhibition (Figure 5) revealed by one of Eshel et al. (2015) experiments. First, we pick the activity of VTA GABA and DA neurons at the last learning trial (*n* = 50), where DA neurons are excited by the cue (CS) rather than by the actual reward (US). Note that in Eshel et al. (2015), DA neurons were still activated at the US timing, which we suppose to be related to their experimental procedure consisting of delivering rewards stochastically (with 90% probability in this experiment). Second, as in Eshel et al. (2015), we simulated GABA photo-inhibition in a time-window (±500 ms) around the reward delivery time (Figure 5A, green shaded area). Considering that ArchT virus expression was partial in GABA neurons and that optogenetic effects do not account quantitatively for physiological effects, the photo-inhibition was simulated for only 20% of our GABA population. This simulated inhibition resulted in a disinhibition of DA neurons activity during laser stimulation (Figure 5B). If the inhibition was 100% efficient on GABA neurons, we assume that experimentally, DA neurons would then burst at high frequencies during the whole period of stimulation.

Inhibiting VTA GABA neurons partially reversed the expectation-dependent reduction of DA response at the US. As proposed by Eshel et al. (2015), our model accounts for the burst-canceling expectation signal provided by VTA GABA neurons.

### 3.3. Effects of Nicotine on RPE Computations in the VTA

We next asked whether we can identify the effects of nicotine action in the VTA during the classical-conditioning task described in Figure 3. We compared the activity of DA neurons at different conditioning trials to their activity after 5 min of 1 μ M nicotine injection, corresponding to physiologically relevant concentrations of Nic in the blood after cigarette-smoking (Picciotto et al., 2008; Graupner et al., 2013). For our qualitative investigations, we assume that α4β2-nAChRs are mainly expressed on VTA GABA neurons (*r* = 0.2) and we study the effects of nicotine-induced desensitization on these receptors.

**Figure 6:**
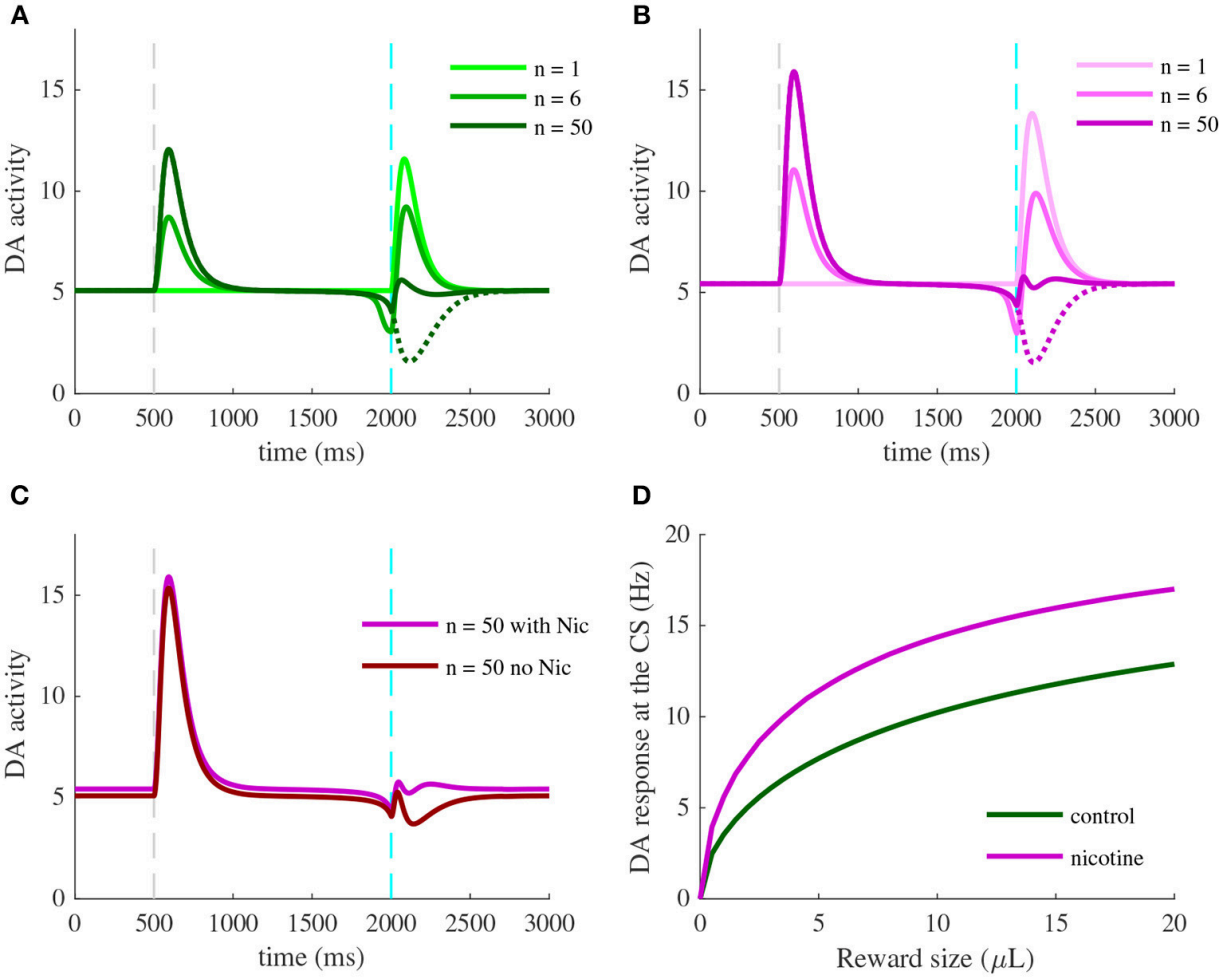
Effects of nicotine on DA activity during classical-conditioning. **(A)** Activity of DA neurons during the pavlovian-conditioning (tone + 4 μL reward) task in three different trials as in Figure 3. **(B)** Same as **(A)** but after 5 min of 1 μM nicotine injection during all conditioning trials. **(C)** DA activity after learning under nicotine (magenta) or in the same condition but when nicotine is removed (dark red). **(D)** Dose-response curves of CS-related burst in DA neurons after learning in control condition (green) or under nicotine (magenta).

Nic-induced desensitization may potentially lead to several effects. First, under nicotine (Figure 6B), DA baseline activity slightly increases. Second, simulated exposure also raises DA responses to reward-delivery when the animal is naive (Figures 6A,B, *n* = 1), and therefore to reward-predictive cues when the animal has learnt the task (Figures 6A,B, *n* = 50). As expected, these effects derive from the reduction of the ACh-induced GABA activation provided by the PPTg nucleus (Figure 3C). Thus, our simulations predict that nicotine would up-regulate DA bursting activity at rewarding events.

What would happen if the animal, after having learned in the presence of nicotine, is not exposed to it anymore (nicotine withdrawal)? To answer this question, we investigate the effects of nicotine withdrawal on DA activity after the animal has learnt the CS-US association under nicotine (Figure 6C), with the same amount of reward (4 μL). In addition to a slight decrease in DA baseline activity, the DA response to the simulated water reward is reduced even below baseline (Figure 6C, dark red). DA neurons would then signal a negative reward-prediction error, consequently encoding a possible perceived insufficiency of the actual reward it usually receives. From these simulations, we could predict the effect of nicotine injection on the dose-response curve of DA neurons to rewarding events (Figure 6D).

Here, instead of plotting DA neuron response to different sizes of unexpected rewards as in Figure 2B, we plot DA response to the CS after the animal has learnt different sizes of rewards (Figure 6D), taking the maximum activity in a 200 ms time-window following the CS onset (Figures 6A,B, dark colors). Thus, when the animal learns under nicotine, the dose-response curve is elevated, assigning an amplification effect of nicotine on dopamine reward-prediction computations. Notably, the nicotine-induced increase in CS-related bursts grows with the increase of reward size for rewards ranging from 0 to 8 μL. Associating CS amplitude to the predicted value (Rescorla and Wagner, 1972; Schultz, 1998), this suggests that nicotine could increase the value of the cues predicting large rewards, therefore increasing the probability of choosing the associated states compared to control conditions.

### 3.4. Model-Based Analysis of Mouse Decision-Making Under Nicotine

**Figure 7:**
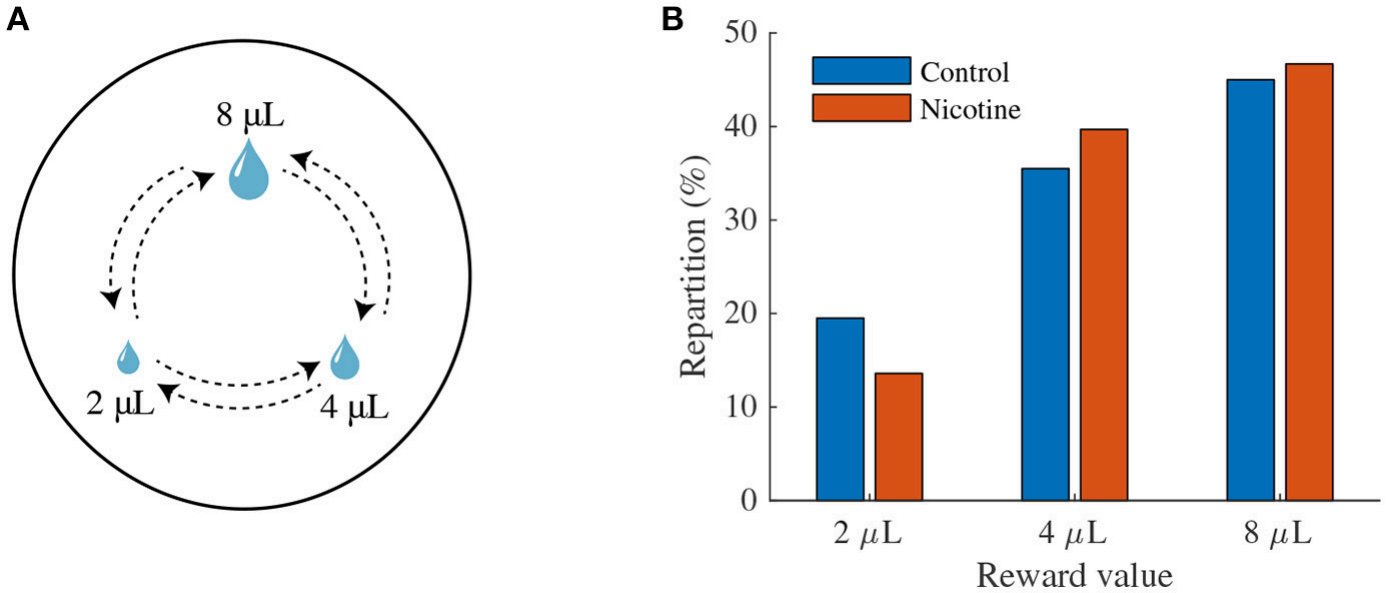
Effects of nicotine on mouse decision-making among reward sizes. **(A)** Illustration of the modeling of the task. Three explicit locations are placed in an open field. Mice receive a reward each time they reach one of the locations. Simulated mice, who could not receive two consecutive rewards at the same location, alternate between rewarding locations. The probability of transition from one state to another depends on the two available options. **(B)** Proportion of choices of the three rewarding locations as a function of reward value (2, 4, 8 μL) over 10,000 simulations in control mice (blue) or nicotine-ingested mice (red).

In order to evaluate the effects of nicotine on the choice preferences among reward sizes, we simulated a decision-making task where a mouse chose between three locations providing different reward sizes (2, 4, 8 μL) in a circular open-field (Figure 7A) inspired by Naudé et al. (2016) experimental paradigm.

Following reinforcement-learning theory (Rescorla and Wagner, 1972; Sutton and Barto, 1998), CS response to each reward size (computed from Figure 6D) was attributed to the expected value of each location. We then computed the repartition of the mouse between the three locations over 10,000 simulations in control conditions or after 5 min nicotine ingestion.

In control conditions, the simulated mice chose according to the location's estimated value (Figure 7B); the mice chose preferentially the locations that provide the greater amount of reward. Interestingly, under Nic-induced nAChRs desensitization, the simulations show a bias of mice choices toward large reward sizes; the proportion of choices for the small reward (2 μL) diminished by about 4%. Thus, these simulations suggested a differential amplifying effect of nicotine for large water rewards.

We can explain these simulation results in Figure 6D, by the fact that nicotine has a multiplicative effect on DA responses at the CS in the interval [0,8] μL compared to control condition. This then leads to a proportionally larger nicotine influence on the larger vs. the smaller rewards. We then expect that such bias would not appear for a set of larger rewards, as the nicotine effect is additive after 8 μL. This is a prediction of this model.

## 4. Discussion

The overarching aim of this study was to determine how dopamine neurons compute key quantities such as reward-prediction errors, and how these computations are affected by nicotine. In order to do so, we have developed a computational modeling approach extending the population activity of the VTA and its main afferents during a simple task of Pavlovian-conditioning. Including both theoretical and phenomenological conceptions, this model qualitatively reproduces several observations on the VTA activity during the task: phasic DA activity at the US and the CS and persistent activity of VTA GABA neurons. It particularly proposes a two-speed learning process of the reward timing and size mediated by the PFC working memory, coupled with the signaling of reward occurrence in the PPTg. Finally, using acetylcholine dynamics coupled with the desensitization kinetics of α4β2-nAChRs in the VTA, we revealed a potential effect of nicotine action on reward perception through up-regulation of DA phasic activity.

### 4.1. Modeling Choices

Multiple studies have proposed a dual-pathway mechanism for RPE computation in the brain (O'Reilly et al., 2007; Vitay and Hamker, 2014) through phenomenological bottom-up approaches. Although they propose different possible mechanisms, they mainly gather several components: regions that encode reward-expectation at the CS, regions that encode actual reward, regions that inhibit dopamine activity at the US, and final subtraction of these inputs at the VTA level. These models usually manage to reproduce the key properties of dopamine-related reward activity: progressive appearance of DA bursts at the CS onset, progressive decrease of DA bursts at the US onset, phasic inhibition when reward is omitted and early delivery of reward.

**Figure 8:**
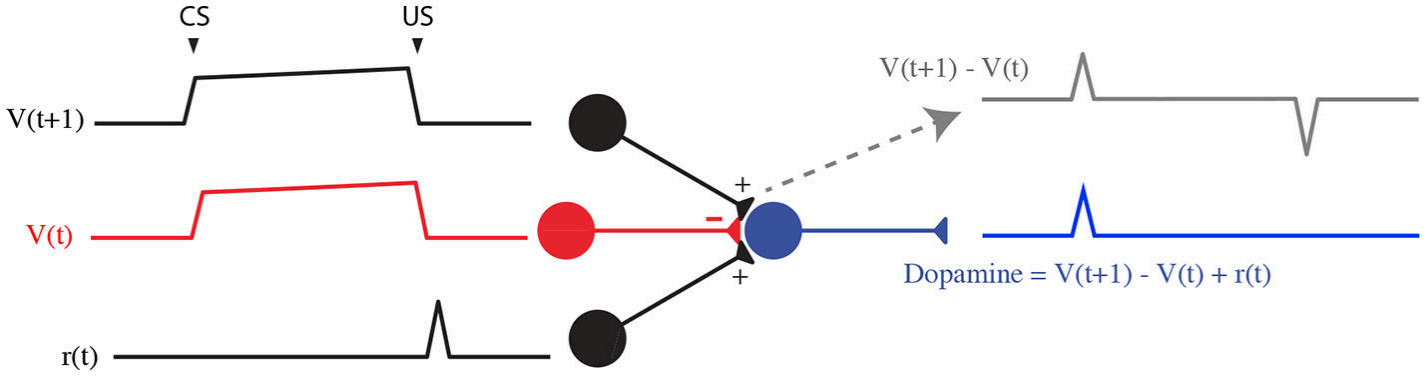
TD learning model (Watabe-Uchida et al., 2017).

Additionally, a top-down theoretical approach as the temporal difference (TD) learning model assumes that the cue and reward cancellation signal both emerge from the same inputs (Sutton and Barto, 1998; Morita et al., 2013). After the task is learned, two sustained expectation signals *V*(*t*) and *V*(*t*+1) subtract each other (Figure 8), leading to the TD error: δ = *r*+*V*(*t*+1)−*V*(*t*). Notably, the temporary shift between both signals induce a phasic excitation at CS and an inhibition at the US.

TD models are reliable to describe many features of dopamine phasic activity and establish a link between reinforcement learning theory and dopamine activity. However, the biological evidence for such specific signals is still unclear.

In our study, we combine these two phenomenological and theoretical approaches to describe the VTA DA activity. Firstly, our simple model relies on neurobiological mechanisms such as PFC working memory activity (Connor and Gould, 2016; Le Merre et al., 2018), PPTg activity (Kobayashi and Okada, 2007; Okada et al., 2009) and mostly VTA GABA neurons activity (Cohen et al., 2012; Eshel et al., 2015) and describe how these inputs could converge to VTA DA neurons. Secondly, at least at the end of learning, we also proposed a similar integration of inputs as in TD models, with two sustained signals that are temporally delayed. We note that in our model, like in the algorithmic TDRL models, late delivery of the reward would lead to a dip in the DA activity at the previously expected reward-time and same for early reward (simulations not shown). Arguably, the late reward response matches experimentally observed phasic DA activity, early reward remains a challenge for the model.

Indeed, the reward expectation signal comes from the same input (PFC): based on recent data on local circuitry in the VTA (Eshel et al., 2015), we assumed that the PFC sends the *V*(*t* + 1) sustained signal to both VTA GABA and DA neurons. Only, via a feed-forward inhibition mechanism, this signal is shifted by VTA GABA neurons membrane time constant τ_G_. Thus, in addition to the direct *V*(*t* + 1) excitatory signal from the PFC, VTA GABA neurons would send the *V*(*t*) inhibitory signal to VTA DA neurons (Figure 8). Adding the reward signal *r*(*t*) provided by the PPTg, our model integrates the TD error δ into DA neurons. However, in our model, and as shown in several studies, CS- and US-related bursts gradually increase and decrease with learning, respectively, whereas TD learning predicts a progressive backward shift of the US-related burst during learning, what is not experimentally observed.

Although we make strong assumptions on VTA reward information integration that may be questioned at the level of detailed biology, it proposes a way to explain how the sustained activity in GABA neurons cancel the US-related dopamine burst without affecting the preceding tonic activity of DA neurons during the CS-US interval. Furthermore, this assumption can be strengthened by our simulation of optogenetic experiment (Figure 5) qualitatively reproducing DA increase in both baseline and phasic activity as found in Eshel et al. (2015).

### 4.2. Reliability of the VTA Afferents

As described above, our model includes two glutamatergic and one GABAergic input to the dopamine neurons, without considering the influence of all other brain areas.

Although the NAc disinhibitory input and the PPTg excitatory input were found to be important de-facto excitatory afferents to the VTA, it remains elusive whether these signals: (1) respectively encode reward expectation and actual reward and (2) are the only excitatory inputs to the VTA during a classical-conditioning task. As well, it is still unclear whether VTA GABA fully inhibit their dopamine neighbors. Here, we assumed that the activity of DA neurons with no GABAergic input was relatively high (*B*_D_ = 18 Hz) in order to compensate the observed high baseline activity of GABA neurons (*B*_G_ = 14 Hz) and get the observed DA tonic firing rate (≃ 5 Hz). This brings up two issues: do these GABA neurons only partially inhibit their dopamine neighbors, for example, just when activated above their baseline? And also, is the inhibitory reward expectation signal mediated by other brain structures as the LHb (Watabe-Uchida et al., 2012; Keiflin and Janak, 2015; Tian and Uchida, 2015)?

In an attempt to answer this question, Tian et al. (2016) recorded extracellular activity of monosynaptic inputs to dopamine neurons in seven input areas including the PPTg. Showing that many VTA inputs were affected by both CS and US signals, they proposed that DA neurons receive a mix of redundant information and compute a pure RPE signal. However, this does not elucidate which of these inputs effectively affect DA neurons activity during a classical-conditioning task.

While other areas might be implied in RPE computations in the VTA, within our minimal model, we used functional relevant inputs to the VTA that were shown to be strongly affected by reward information based on diverse recurrent studies in the last decades: the working-memory activity in the PFC integrating the timing of reward occurrence (Durstewitz et al., 2000; Connor and Gould, 2016), the dopamine-mediated plasticity in the NAc via dopamine receptors (Morita et al., 2013; Yagishita et al., 2014; Keiflin and Janak, 2015), the PPTg activation at the reward delivery (Okada et al., 2009; Keiflin and Janak, 2015). Notably, in most of our assumptions, we rely on experimental data that studied neuronal activity of mice performing a simple classical-conditioning task (reward delivery following conditioning cue with no instrumental actions required). In line with this modeling approach, further optogenetic manipulations implying photo-inhibition as in Eshel et al. (2015) would then be required to study the exact functional impact of the PFC, the NAc and the PPTg on dopamine RPE computations during a simple classical conditioning task.

### 4.3. Learning of Reward Expectation in the Corticostriatal Pathway

Our model proposes a specific scenario for PFC-NAc pathway integration of both reward timing and expectation, its biological plausibility is a significant discussion point.

The reward timing learning mechanism exposed in Equation (6) was inspired from Luzardo et al. (2013), who proposed that reward delivery timing can be learnt by adapting the drift rate of a neural accumulator whose firing rate is expected to reach a specific value at the reward delivery timing. If the reward occurs earlier than expected, the slope of this accumulator is increased. However, if the accumulator reaches its value before US timing, its slope is decreased. Therefore, the rule uses an error signal that is based on time discrepancy between the neural activity reaching a threshold and the reward. Here, we used the same error signal Δ*t*, but the affected parameter is the recurrent excitation strength *J*_PFC_ and the neural activity dynamics is not an accumulator but an attractor.

We further assumed that this update mechanism could be linked with a potential dopamine-mediated modulation in the PFC (Puig et al., 2014; Popescu et al., 2016) such that ν_PFC_ rapidly decreases (transition from the active to the rest attractor) at the US timing. Although this dopamine-mediated timing representation hypothesis remains to be directly investigated experimentally, several lines of experimental evidence could support it. First, it is widely accepted that the PFC activity does represent timing information relevant to cognitive tasks through sustained firing activity (Curtis and D'Esposito, 2003; Morita et al., 2012; Xu et al., 2014; Connor and Gould, 2016). Second, it has been shown that dopamine enables the induction of spike-timing dependent long-term potentiation (LTP) in layer V PFC pyramidal neurons by acting on D1-receptors (D1R) on excitatory synapses and D2-receptors on local PFC GABAergic interneurons to suppress inhibitory transmission (Xu and Yao, 2010). Moreover, administration of D1 and D2-receptors antagonists in the PFC during learning has been found to impair discrimination of behaviorally relevant events (Popescu et al., 2016).

Additionally, several DA-RPE models proposed a role for the PFC in providing an eligibility trace required in TD-learning algorithms (O'Reilly et al., 2007; Morita et al., 2012, 2013), considering working-memory representation as crucial in trace conditioning paradigms. Particularly, a specific PFC neuron population, called corticopontine/pyramidal tract (CPn/PT) cells, was assumed by Morita et al. (2012) and Morita et al. (2013) to represent the previous state *s*(*t*) or action *a*(*t*) as sustained activity due to the strong recurrent excitatory connections. Note however, that in their model, this signal was supposed to be inhibitory on DA neurons, as it was designed to go through the indirect cortico-striato-VTA pathway, which were assumed to represent *V*(*t*) (Figure 8). Here, we consider the sustained PFC signal to be excitatory by acting through the direct cortico-striato-VTA pathway, and that the inhibitory component was held by local VTA GABA neurons. In sum, these studies suggested us to consider the PFC as the main timing integrative component of dopaminergic RPE computations through DA-mediated plasticity.

It would be interesting to consider how CS-related sensory inputs (*w*_CS_ in the model) can be amplified with learning by sensory neuroplasticity, in addition to the dopamine-mediated effect on cortical recurrent connections (Equation 6). This possibility was tested in our model: by updating *w*_CS_ in addition to *J*_PFC_ (PFC recurrent connection strength), PFC neuron activity reaches the Up state earlier. It would then accelerate learning in the PFC but end up with the same maximal activity (obtained at *n* = 6 in Figure 3A). Thus, we see that considering sensory representation plasticity is relevant in our context, however it would add another variable to our model without changing the qualitative activity of our neuronal populations. We thus chose not to include these considerations in our minimal model explicitly.

Finally, it is still unclear how DA-mediated plasticity in the striatum could enable the learning of value by striatal neurons. In support of this assumption, it has been suggested that D1R signaling favors synaptic potentiation whereas D2R signaling has the opposite effect (Shen et al., 2008). Moreover, it has been found that in absence of behaviorally important stimuli, DA neurons fire tonically to maintain striatal DA concentrations at levels sufficient to activate D2R, but not low affinity-D1R (Gonon, 1997). We thus considered that dopamine-mediated corticostrial plasticity depended on DA phasic signaling on D1R containing-Medium spiny neurons (MSNs) leading to the activation of the direct excitatory (disinhibitory) pathway to the VTA. Future studies following Morita et al. (2013) modeling work could focus on the respective implication of D1 and D2R MSNs in corticostriatal plasticity during learning.

### 4.4. Nicotine-Induced Effects on nAChRs During Learning

As mentioned above, our local VTA circuit model including nAChRs-mediated current dynamics takes its cue from the minimal model introduced in Graupner et al. (2013). This model was later used to explain effects of pharmacological manipulations on nicotinic receptors (Maex et al., 2014), phasic DA response to nicotine injections (Tolu et al., 2013) and the potential impact of receptor up-regulation following prolonged exposure to nicotine (Dumont et al., 2018). In the original work, Graupner et al. (2013) examined, using computational models, under what conditions (e.g., endogenous cholinergic tone and inputs) one could explain the nicotine-evoked increases in dopamine cell activity and dopamine outflow. To do so, the relative expression of the receptors was parameterised between the DA neurons and the VTA GABA interneurons. In the former case, nicotine would act directly to excite the DA neurons by activating the receptors; in the latter, nicotine would disinhibit the dopamine neurons to increase their firing rate by receptor desensitization. In short, they concluded that both schemes are possible, yet under different endogenous ACh conditions. The direct excitation scheme requires a low Ach tone, while the disinhibition case would yield a robust DA increase under a high ACh tone. We followed the disinhibition scheme since we reasoned that it would be more relevant to behavioral situations where ACh tone is high - notably during motivation-guided behavior and reward seeking (Picciotto et al., 2008, 2012). Had we considered the direct excitation scheme, certainly the outcomes of our model would be different. Notably, we reason that nicotine would lead to an immediate boost of RPE upon delivery, and then depress the RPE for subsequent CS-US pairings. Whether this is compatible with experimentally observed effects and behavior remains to be explored in subsequent studies.

Desensitization of α4β2-nAChRs on VTA GABA neurons following nicotine exposure results in increased activity of VTA DA neurons (Mansvelder et al., 2002; Picciotto et al., 2008; Graupner et al., 2013). Through the associative-learning mechanism suggested by our model, nicotine exposure would therefore up-regulate DA-response to rewarding events by decreasing the impact of endogenous acetylcholine on VTA GABA neurons provided by the PPTg nucleus activation (Figure 6). Together, our results propose that nicotine-mediated nAChRs desensitization potentially enhances the DA response to environmental cues encountered by a smoker (Picciotto et al., 2008).

Indeed, here, we considered that the rewarding effects of nicotine could be purely contextual: nicotine ingestion does not induce a short rewarding stimulus (US), but an internal state (here, after 5 min of ingestion) that would up-regulate smoker perception of environmental rewards (the taste of coffee) and consequently, when learned, the associated predictive cues (the view of a cup of coffee). While nicotine self-administration experiments considered nAChRs activation as the main rewarding effect of nicotine (Picciotto et al., 2008; Changeux, 2010; Faure et al., 2014), our model focuses on the long-term (min to hours) effects of nicotine that a smoker usually seeks, that interestingly correlates with desensitization kinetics of α4β2-nAChRs (Changeux, 2010).

However, the disinhibition hypothesis on nicotine effects in the VTA remains debated. Although demonstrated *in vitro* (Mansvelder et al., 2002) and *in silico* (Graupner et al., 2013), it is still not clear whether nicotine-induced nAChRs desensitization preferentially acts on GABA neurons within the VTA *in vivo*. This would depend on the ratio of α4β2-nAChRs expression levels *r* but also on the preferential VTA targets of cholinergic axons from the PPTg. While we gathered both components into the parameter *r*, recent studies found that PPTg-to-VTA cholinergic inputs preferentially target either DA neurons (Dautan et al., 2016) or GABA neurons (Yau et al., 2016). Notably, accounting for the relevance of Yau et al. (2016) experimental conditions—photo-inhibition of PPTg-to-VTA cholinergic input during a Pavlovian-conditioning task—we chose to preferentially express α4β2-nAChRs on GABA neurons (*r* = 0.2).

It is worth considering that the nicotinic receptors implied in this model are widely expressed throughout the brain. Notably, these are expressed in the PFC on both interneurons and pyramidal neurons, and direct effects of nicotine on the PFC activity has been shown (Picciotto et al., 2012; Poorthuis et al., 2013), together with an impact on VTA DA neurons. Nevertheless, previous work suggests that β 2-containing nAChRs in the VTA are crucial for the animals ability to require stable nicotine self-administration and control the firing patterns of the VTA dopamine neurons (Maskos et al., 2005; Changeux, 2010; Faure et al., 2014). Clearly, our model does not give a full picture of how nicotine may affect learning of motivated behaviors as it does not yet explore the effect of nicotine on cortical dynamics. While we believe this to be a fruitful future direction of study, we would claim that our model gives a minimal sufficient description for the experimental observation that nicotine appears to preferentially boost large vs. small rewards choices through affecting specifically the RPE calculations in the VTA.

### 4.5. Predicted Potential Consequences of Nicotine Exposure on Human Decision-Making

In our behavioral simulations of a decision-making task (Figure 7), we report that nicotine exposure could potentially bias mice choices toward big rewards. Recent recordings from Faure and colleagues (unpublished data) showed a similar effect of chronic nicotine exposure, with mice showing increasing choices for locations with 100% and 50% reward probabilities at the expense of the location with 25% probability. In this line, future studies could investigate the effects of chronic nicotine on VTA activity during a classical conditioning task as presented here (Figure 6) but also on behavioral choices according to reward size (Figure 7).

In sum, our minimal model has shown that nicotine would have a double effect on the dopamine signaling of RPE. First, it reopens the window on previously learned rewarding stimuli, where positive error signals are again apparent after the animal has learnt the CS-US association under control conditions (Figure 6). Second, when we examine the effects of nicotine on reward-size choices, we see that the new nicotine-released phasic DA signals are disproportionally boosted for large rewards. Hence, we may speculate that nicotine could result in a pathologically increased reward sensitivity to large vs small rewards in decision making and behavior. Such reward sensitivity can lead to an apparent prevalence of exploitative behavior. In other words, if the nicotine-exposed animal overestimate the value of choices disproportionally to others, and base its choices on these values, it would essentially focus on its choices on the over-biased large reward choice at the expense of the under-biased small reward choice. Furthermore, some data indicate that in smokers, delay discounting is abnormal, but not for small immediate and very large delayed rewards (Addicott et al., 2013). Here again, one may associate reward sensitivity as a vehicle, and the mechanisms we suggest playing a role. Nicotine abnormally boosts the value (utility) of the very large reward, relatively depressing the small reward and hence biasing the choice toward the delayed (large) reward, which would appear to resist discounting.

Speculatively, in an environment with high reward volatility, such nicotine-induced exploitation would look like an apparent behavioral rigidity. Several human studies have indeed suggested increased reward sensitivity in smokers (Naudé et al., 2015) and an increase in exploitation vs exploration in smokers versus controls (Addicott et al., 2013). Our model would predict that such behavior would arise from the boosted dopaminergic learning signals due to nicotine action on the VTA circuitry. This is of course with the caveat that in our model we did not discuss the multiple brain decision systems that intervene in real life, but focused exclusively on VTA computations.

The idea that dopamine neurons signal reward-prediction errors has revolutionized the neuronal interpretation of cognitive functions such as reward processing and decision-making. While our qualitative investigations are based on a minimal neuronal circuit dynamics model, our results suggest areas for future theoretical and experimental work that could potentially forge stronger links between dopamine, nicotine, learning, and drug-addiction.

## Author Contributions

ND designed research, performed research, wrote the manuscript. BG designed research, advised ND, obtained funding, wrote the manuscript. VM obtained funding, wrote the manuscript.

### Conflict of Interest Statement

The authors declare that the research was conducted in the absence of any commercial or financial relationships that could be construed as a potential conflict of interest.
