# Supplementary material for: Minimal Circuit Model of Reward Prediction Error Computations and Effects of Nicotinic Modulations
Source: Front Neural Circuits. 2019 Jan 8;12:116. doi: 10.3389/fncir.2018.00116 (PMC6336136; doi:10.3389/fncir.2018.00116)
Supplement: Supplementary file 1 [file Data_Sheet_1.pdf]

SUPPLEMENTARY INFORMATION

Parameter	Description	Value	Reference
**α4β2-nAChR**			(Graupner et al., 2013)
EC_{50}	half-maximum conc. of activation (ACh)	30 μM	
α	potency of Nic to evoke response	3	
n_a	Hill coefficient of activation	1.05	
IC_{50}	half-maximum conc. of desensitization by Nic	0.061 μM	
n_d	Hill coefficient of desensitization	0.5	
τ_a	activation time constant	5 msec	
K_τ	half-maximum conc. of desensitization time const.	0.11 μM	
n_τ	Hill coefficient of desensitization time constant	3	
τ_{max}	maximal desensitization time constant	10 min	
τ_0	minimal desensitization time constant	500 msec	
**Network**			
x	reward size	1-20 μL	(Eshel et al., 2015)
w_{CS}	strength of CS signal	8	here
τ_{D}	membrane time constant of DA population	30 ms	(Graupner et al., 2013)
τ_{G}	membrane time constant of GABA population	30 ms	(Graupner et al., 2013)
τ_{PFC}	membrane time constant of PFC population	100 ms	(Gerstner et al., 2014)
τ_{a}	adaptation time constant	1000 ms	(Gerstner et al., 2014)
τ_{PPTg}	membrane time constant of PPTg population	80 ms	(Okada et al., 2009)
w_{G}	strength of GABA input to DA	1	(Graupner et al., 2013)
$w_{\text{PFC-D}}$	strength of PFC input to DA	variable	
$w_{\text{PFC-G}}$	strength of PFC input to GABA	variable	
J_{PFC}	strength of PFC recurrent connections	variable	
$w_{\text{PPT-D}}$	strength of PPTg Glu input to DA	0.8	(Yoo et al., 2017)
$w_{\text{PPT-G}}$	strength of PPTg Glu input to GABA	0.2	(Yoo et al., 2017)
$w_{\alpha4\beta2}$	strength of nAChR activation	15	here
w_{ACh}	maximal ACh conc. from PPTg	1 μM	(Graupner et al., 2013)
B_{D}	baseline firing rate of DA (without input)	18 Hz	(Eshel et al., 2015)
B_{G}	baseline firing rate of GABA	14 Hz	(Eshel et al., 2015)
B_{PPTg}	baseline firing rate of PPTg	2 Hz	(Okada et al., 2009)
r	balance of α4β2 nAChRs	0.2	(Mansvelder et al., 2002)
c	strength of adaptation in PFC population	0.6	here
α_{P}	learning rate of PFC recurrent weight	0.2	here
α_{S}	learning rate of cortico-striatal weight	0.0005	here

Table 1. Model parameters The parameters in the model were chosen qualitatively in order to account for most of experimental data from different studies (references) with relative accuracy. The α4β2-containing nAChR parameters were directly taken from (Graupner et al., 2013), whereas the network parameters were qualitatively adapted from different studies. When no data could be related, some parameters were arbitrarily fixed (here).

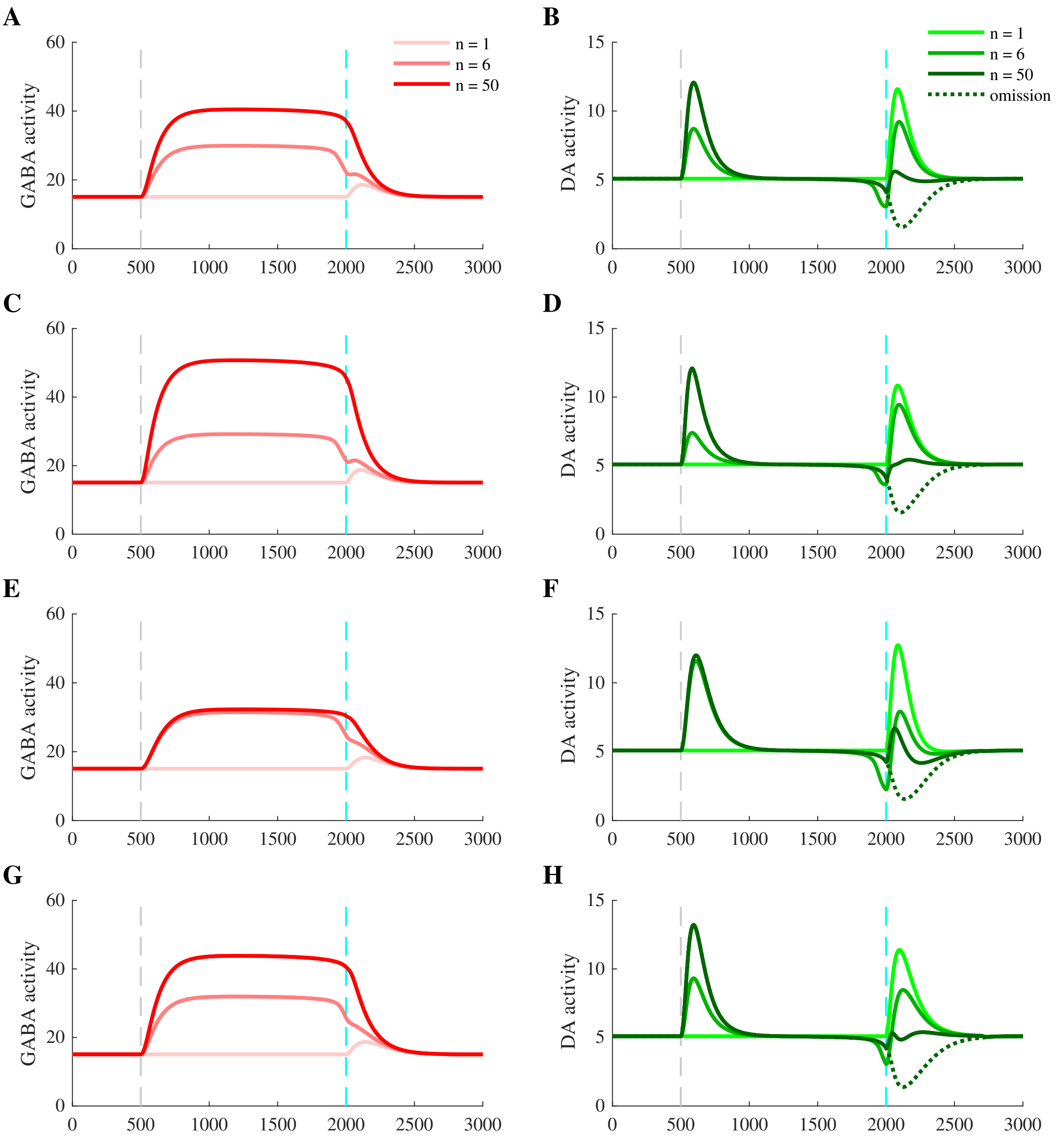

Figure S1. Sensitivity of VTA activities to parameter variations

Simulated mean activity (Hz) of VTA GABA and DA neuron populations during a pavlovian-conditioning task, where a tone is presented systematically 1.5 s before a water reward (4 μL) (see Fig. 3 for full description). Three different trials are represented: the initial conditioning trial ($n = 1$, light colors), an intermediate trial ($n = 6$, medium colors) and the final trial ($n = 50$, dark colors) and when reward is omitted after learning (dotted lines). Vertical dashed grey and cyan lines represent CS and US onsets, respectively. PPTg and GABA time constants were varied for each simulation:
(A,B) τ_{PPTg} = 80 ms, τ_{G} = 30 ms (default time constants),
(C,D) τ_{PPTg} = 80 ms, $\boldsymbol{\tau_{\mathrm{G}}}$ **= 20 ms**,
(E,F) τ_{PPTg} = 80 ms, $\boldsymbol{\tau_{\mathrm{G}}}$ **= 50 ms**,
(G,H) $\boldsymbol{\tau_{\mathrm{PPTg}}}$ **= 100 ms**, τ_{G} = 30 ms
